# Short-term memory for spatial, sequential and duration information

**DOI:** 10.1016/j.cobeha.2017.05.023

**Published:** 2017-10

**Authors:** Sanjay G. Manohar, Yoni Pertzov, Masud Husain

**Affiliations:** 1 Dept Experimental Psychology and Nuffield Dept of Clinical Neuroscience, University of Oxford, United Kingdom; 2 Dept of Psychology, The Hebrew University of Jerusalem, Israel

## Abstract

Space and time appear to play key roles in the way that information is organized in short-term memory (STM). Some argue that they are crucial contexts within which other stored features are embedded, allowing binding of information that belongs together within STM. Here we review recent behavioral, neurophysiological and imaging studies that have sought to investigate the nature of spatial, sequential and duration representations in STM, and how these might break down in disease. Findings from these studies point to an important role of the hippocampus and other medial temporal lobe structures in aspects of STM, challenging conventional accounts of involvement of these regions in only long-term memory.

## Highlights

• Analog report methods provide novel insights on STM for space and time.
• Space and time may be used to bind features in STM.
• The hippocampus is involved in object-location binding in STM.

**Current Opinion in Behavioral Sciences** 2017, **17**:20–26

This review comes from a themed issue on **Memory in time and space**

Edited by **Lila Davachi** and **Neil Burgess**

For a complete overview see the Issue and the Editorial

Available online 20th June 2017

**http://dx.doi.org/10.1016/j.cobeha.2017.05.023**

2352-1546/© 2017 The Authors. Published by Elsevier Ltd. This is an open access article under the CC BY license (http://creativecommons.org/licenses/by/4.0/).

## Introduction

Research on STM (storage of information over a few seconds) and working memory (WM, manipulation of information held in STM) has gained new impetus over the last few years. One important debate that has fueled this interest centers on the architecture of short-term memory. Classical views of STM capacity have considered it to be both quantized and limited to a small number of discrete memory ‘slots’, each of which contains a single object, with all its features bound veridically together. By contrast, recent investigations have provided evidence for a limited representational medium, which can be flexibly distributed between objects, without any fixed item capacity limit [1].

This view has emerged from the introduction of continuous, analog report methods that require participants to reproduce from memory their recollection of a feature of the item stored, rather than to state in binary fashion whether an item had been present or not in the memory array. These new behavioral techniques have had a strong impact on the field, challenging some influential views of STM and WM, how attention interacts with STM and even the role of the hippocampus in STM. In this review, we focus on how these new methods have provided new tools to probe brain mechanisms underlying STM for spatial location, sequences, and temporal durations (for other recent perspectives, see Refs. [1, 2, 3, 4]).

**Figure 1:**
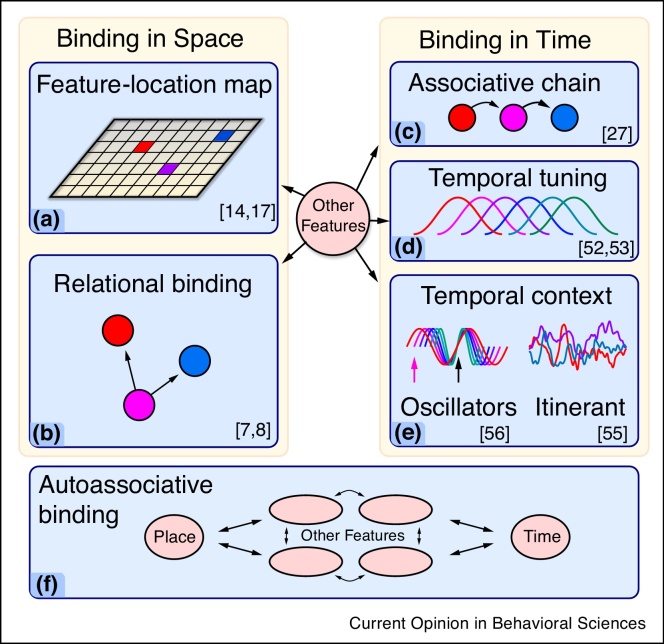
Space and time in short term memory. Features encoded separately must be brought together into objects, in order to support cued recall. This binding could rely on several mechanisms. Both space and time can be considered as independent universal contexts for binding features together. *Left:***(a)** Features could be bound by pairing each feature with a particular location in space. **(b)** Alternatively, pairs of objects might be connected by configural information, with locations encoded primarily in terms of spatial directions. In this case, an object’s location is stored in terms of its direction relative to other objects in memory. *Right:* Features can be grouped in terms of their co-occurrence in time. **(c)** A simple model of temporal ordering links each object representation to its successor. **(d)** Alternatively, events might be attached to a time-specific code, in which distinct representational units are active at different moments in time. **(e)** Recent models of temporal binding postulate a high-dimensional time code composed of multiple time-varying traces which, together, indicate the time an event occurs. *Bottom:***(f)** Time and space could also be considered as features in their own right. In this scenario, time and place are on equal terms with other features of the object.

Theoretical considerations of the role of space and time in STM/WM have led to two distinct views. On one account, space and time are simply features or attributes – similar to color or shape – that all get bound together (*e.g.*, as an ‘object file’). The alternative view is that space and time are fundamental ‘contexts’, acting as a medium *within* which all other features occur, and other features can bind only to these spatiotemporal contexts. Several different mechanisms have been proposed to support spatial and temporal contexts (Figure 1).

## STM for space

In real life situations, we often use information about the space around us, even when it is no longer perceived by our senses. Behaving effectively in dynamic settings, where we or other agents are on the move, often requires the use of STM for spatial locations [5]. For example, when people prepare tea in a kitchen, their gaze often shifts precisely towards (remembered) targets, such as the kettle or cup, which lie outside their field of view [5].

### Slots vs resources and biases in memory for location

The precision of STM for space can be assessed by asking participants to localize in space where a specific stimulus was displayed. The slot model predicts that recall behavior should plateau when the number of items goes beyond the number of slots available. A recent study using pointing movements showed, however, that recall variability for items in memory simply increases monotonically from 1 to 8 items, incompatible with such a fixed capacity, quantized model [6^•^]. Location memory also seems to be systematically distorted. Such biases can shed light on how space is represented in STM. When participants are required to reproduce a location from memory, estimates are often shifted away from the outer edges of a defined space as well as from internal axes of symmetry. These findings suggest that memory reports combine information about stimulus location with information about the dominant frame of reference that people apply to the space [7]. Such biases become more pronounced the longer a stimulus is held in memory [8] which indicates that the bias results, at least partly, from maintaining the information in STM rather than from a bias in perception.

### Object-location binding

Spatial location information on its own is not very useful to hold in memory. We typically need to know “What was where?” Thus, object information has to be bound to remembered locations. Position information appears to have a privileged role in STM, with different features belonging to an object (*e.g.*, orientation and color) seemingly bound to each other via their shared position. Items that share their position are more likely to be mixed up in STM reports, but not items that share the same color [9]. Non-target items located closer to the memory target also interfere with it more often than items that are distant [10]. Moreover, longer retention intervals lead to worse memory performance mainly due to reporting items in the wrong location [11••, 12].

According to location uncertainty theory [13] such confusions in perception (*i.e.*, illusory conjunctions) are caused by uncertainty concerning the locations of objects in space. A recent model of STM explicitly described a possible mechanism for explaining these ‘swap’ errors in visual STM [14^••^]. It incorporates a two-layer neural network, in which one layer represents memory contents (*e.g.*, orientations or colors), and the other represents their contexts. Context could either be time or space, and binding to context is maintained in two dimensional ‘binding space’. Cue-based retrieval starts from activating the representation of the cued context in context space, which generates a distribution of activation in memory content space through the bindings in binding space. Each feature receives activation according to the strength of its binding to the context cue. Thus, the feature of the item that had been in the cued context is likely to be activated most strongly. Because of the width of the activations in binding space and context dimension, a retrieval cue is also likely to reactivate memory content of other items in the memory. Thus, noise in the system could lead to reporting features of other items in memory—swap errors (for a more detailed neural architecture of binding in STM see Ref. [15]). Over and above swap errors, concurrently remembered items may also have push–pull effects on each other [16, 17], which can be predicted by continuous attractor models. In these models, spatial and nonspatial features of an item are maintained during the delay period through persistent activity, but are perturbed by noise leading to drift in the remembered features [18].

### Neuroscience of spatial STM

**Figure 2:**
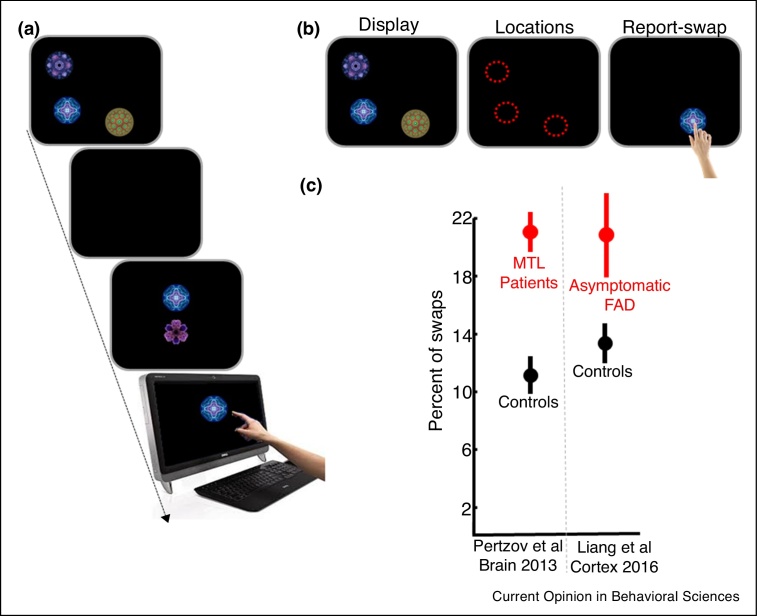
Object-location binding in short-term memory. **(a)** ‘What was where?’ task. One or three fractals were simultaneously presented in pseudo-random locations. Following a delay, a two alternative forced choice between one of the displayed fractals and a foil was presented. Participants were required to ‘drag’ the previously presented fractal on the touch screen to its remembered, original location on the screen. **(b)** Swap or misbinding errors are defined as trials in which the correct item was selected but localized precisely near one of the original locations of the other fractals in the memory array (*e.g.*, rightmost panel). **(c)** Patients with compromised hippocampus function (VGKC patients and asymptomatic Familial Alzheimer’s Disease) exhibit abnormally frequent swap errors.

Recent findings have challenged the view that the hippocampus plays a role in long-term memory but not STM. Binding of objects to their position in STM is impaired in neurological conditions that involve the hippocampus. Patients with an immune-mediated limbic encephalitis which appears to target medial temporal lobe structures including the hippocampus are specifically impaired in object-location binding over short retention intervals, but not in remembering the position or identity on their own [19]. This result was obtained using a new “What was where?” task which provides a continuous, analog report of memory for location on a touchscreen (Figure 2). An identical deficit was recently reported using the same task in individuals with pathological mutations in *Presenilin*-1 or amyloid precursor protein genes for familial Alzheimer's disease (FAD) [20^•^]. The study revealed a strong association between decreased hippocampal volume across FAD participants and deficits in object-location binding.

Similar results pointing to difficulties in object-location binding in STM have been reported in patients with hippocampal damage with a variety of etiologies, including herpes simplex encephalitis, anoxia and limbic encephalitis [21, 22]. Patients with impaired medial temporal lobe pathology, specifically involving the hippocampi, were severely impaired at scene discrimination when a significant demand was placed on short term retention of complex spatial information in viewpoint independent representations [23, 24]. Moreover, multivoxel pattern analysis of human functional imaging data supports the view that the hippocampus plays a role in binding object and location information even over short intervals [25], especially when the memory task is difficult [26]. Identity and location information was observed in the patterns of activity of perirhinal and parahippocampal cortex respectively, whereas activity patterns in the right anterior hippocampus across encoding and delay periods was predictive of accurate short-term memory for object–location relationships [25]. Recent experiments in rodents have also reported findings which suggest hippocampal-prefrontal interactions supporting STM for location or objects in locations are important for mediating encoding or retrieval of context-dependent memories over short durations [27, 28••].

## STM for time

### STM for temporal sequences

Sequential or temporal order provides an alternative to spatial location for addressing or indexing multiple pieces of information. Like spatial location, it may also facilitate binding of features into objects. Holding a sequence in STM produces both recency and primacy benefits, with specific patterns of transposition and intrusion errors [29]. One of the oldest explanations for these is associative chaining, in which pairs of contiguous items are neurally associated [30]. More recent explanations have proposed more nuanced neural mechanisms discussed below.

### Neuroscience of temporal sequences

Neurons that are selectively activated for particular serial positions in a sequences are present in many areas of the frontal cortex of monkeys [31] and in rat hippocampus [28]. Such position-selectivity is notoriously hard to pin down in human functional imaging experiments, though the rostrolateral prefrontal cortex and anterior temporal lobe have been implicated [32]. An alternative to position-selectivity is to represent a sequence hierarchically, as ‘chunks’. Although it is often studied in motor control, chunking ability correlates strongly with the ability to update WM [33].

Although the hippocampus is often considered central in binding spatial and temporal contexts in longer-term episodic memory, it is increasingly recognized that it contributes to short-term recall also [22]. In long term memory, populations of hippocampal neurons can represent elapsed time due to their tendency to activate in sequences [34]. Such sequential activation patterns can be observed using MEG [35], and arise in rapid sequences even without overt behavior [36]. Separate to sequential activation, temporal context information of learned sequences may be represented in parahippocampal cortex [37]. Further work is needed to establish if these mechanisms also contribute to STM.

One proposed way of encoding sequences is by rapid sequential activation of representations, in order, with a whole sequence being repeated every 200 ms [38]. According to this account, the rapid cycling between representations (at a gamma frequency, *i.e.*, every 10–40 ms) allows memories of sequences to be held online in STM. This strong hypothesis has received some neurophysiological support, with neurons firing most frequently just before the trough phase of theta oscillations during short term retention tasks in visual cortex [39]. MEG data in humans supports this, demonstrating that peak gamma amplitude shifts to distinct theta phases during encoding of sequential memory items [40].

### Does time cause short term memories to decay?

STM of an item has been thought to remain stable for as long as attention is sustained. Elapsing time has often been considered responsible for the decay of information over a retention interval, with evidence supporting models based on rehearsal [41], or drift [42] and extinction [43] in neural representations. Against this, it has been shown that memory decay can be reduced if the gap between trials (when nothing is happening) is much longer than the retention interval [44]. This suggests that representations do not simply decay over time, but rather their accessibility depends on interference from neighboring events in time. Events that are closer in time may be less distinct, and thus recalled less precisely, due to interference from the superposition of associations [14,45]. This is consistent with the finding in auditory digit recall that events in the retention interval are timed less precisely as load increases [46].

### STM for durations

A special case of sequence memory arises when time intervals themselves must be remembered. Most studies that investigate memory for sequences of durations test our ability to discriminate rhythms, that is, sequences of durations that are integer multiples of a discrete, quantised beat [47]. These have demonstrated a soft limit to the number of durations that can be remembered which is much greater than for non-rhythmic sequences. Rhythm may predispose us to employ discrete categorical strategies for representing time, by emphasizing the relation between sequential intervals, and thereby using a more economical code. Non-rhythmic time sequences, on the other hand, may recruit different neural mechanisms [48]. Perceiving rhythm also leads to phase-dependent facilitation for many aspects of auditory perception and cognition [49]. Rhythm-perceptual effects may lead to more economical storage of intervals at the expense of precision [50], similar to ‘lossy compression’, configural or familiarity effects observed in visual memory [51•, 52].

### Neuroscience of STM for temporal duration

How might neurons encode time durations in memory? Three classes of time encoding have been proposed: ***activity-level coding*** in which the average population firing rate correlates with duration, ***channel-based codes*** in which neurons are selective for different durations, and ***phase-state codes*** in which time-varying activity across the population indicates the duration indirectly, through the phases of individual neurons.

In activity-level codes, a single time interval could be reproduced by allowing neural activity to gradually decrease during the encoding period. At the end of the interval, the final level of activity then determines the subsequent rate-of-rise of an accumulator [53]—somewhat like a pendulum that swings back to the height it was released from. To hold multiple durations, a series of such neuronal populations would be required [54], coordinated by similar processes as those used in visual or verbal WM.

The second class of proposed mechanisms involve an array of time-sensitive channels, each of which is activated by time intervals of a particular duration. Individuals are less sensitive to durations after adapting to repeatedly hearing a fixed duration, analogous to adaptation to visual orientations and spatial frequencies [55]. These adaptation effects are cross-modal, suggesting the presence of domain-general timing channels. Accordingly, single neurons with duration-selectivity have been observed in prefrontal cortex, for durations up to 4 s [56]. Such duration-selective channels, analogous to classical visual and auditory feature domains, may allow durations to be remembered in a similar way to other sensory features. In line with this, similar capacity limits, such as set-size, serial order and pre-cueing effects are evident when remembering durations [57].

The third class includes several recent models of time memory that harness the phase states of individual neurons. Population clock models posit that neural ensembles transition through a sequence of states in a probabilistic manner to produce accurate timing [58]. Alternatively, coincidences of noisy cortical oscillations may be detected by striatal neurons, rendering them sensitive to ‘beats’ that occur after a learned interval [59]. Functional imaging findings suggest that sensorimotor thalamocortical-basal ganglia pathways may subserve the more complex aspects of temporal cognition [60, 61], providing inputs for individuating event durations by the hippocampus [62]. Indeed STM may be central in producing an interval, because some form of counter needs to be maintained online during the produced interval [63^•^]. Conversely, individuating items in STM might utilize the same temporal context cues as interval timing, an idea supported by correlations between memory performance and temporal discrimination performance [64]. Interval timing and STM might thus be two modes of operation of the same neural system [63^•^].

## Conclusion

Both space and time facilitate object binding in STM/WM. Several different mechanisms have been proposed to explain how spatial and temporal information are stored, each with corresponding neural models. Physiological evidence to date has found support for some of these proposals. An important task for future research will be to examine whether these mechanisms are simultaneously employed during STM/WM tasks, and to what extent they overlap, both functionally and neurally.

## Conflict of interest statement

Nothing declared.

## References and recommended reading

Papers of particular interest, published within the period of review, have been highlighted as:

• of special interest
•• of outstanding interest
